# Association between trouble sleeping and cataract in US adults: a cross-sectional study

**DOI:** 10.3389/fmed.2026.1535667

**Published:** 2026-04-23

**Authors:** Jun Huang, Yulan Zhang, Chao Wu, Chen Wu, Yifan Wu, Feiran Wang, Lu Shi

**Affiliations:** Department of Ophthalmology, The Second Affiliated Hospital, Nanchang University, Nanchang, China

**Keywords:** cataract, cross-sectional study, national health and nutrition examination survey, sleep disorder, trouble sleeping

## Abstract

**Objective:**

To investigate the association between trouble sleeping and cataract in US adults, given the increasing global burden of cataracts and the high prevalence of sleep problems.

**Methods:**

This cross-sectional study analyzed data from 2,601 adults aged ≥40 years in the National Health and Nutrition Examination Survey (2005–2008). Trouble sleeping was assessed through self-reported diagnosis, and cataract status was determined by self-reported history of cataract operation. Multiple logistic regression models were adjusted for sociodemographic factors, lifestyle variables, and comorbidities.

**Results:**

Participants with trouble sleeping had significantly higher odds of cataracts (OR = 2.50, 95% CI: 1.35–4.62, *p* = 0.011) after adjusting for all covariates. Subgroup analyses confirmed the robustness of this association across different demographic and clinical characteristics, with interaction tests indicating no significant effect modification between subgroups.

**Conclusion:**

Trouble sleeping was significantly associated with increased odds of cataracts in US adults. These findings suggest potential value in incorporating sleep health into cataract prevention strategies. Future longitudinal studies are needed to confirm causality, explore underlying mechanisms, and evaluate the impact of sleep interventions on cataract risk.

## 1 Introduction

Cataract remains the leading cause of preventable blindness worldwide. Due to global population aging and increasing life expectancy, the number of individuals affected by cataract-related visual impairment is projected to reach 40 million by 2025 (1). However, substantial global disparities exist in cataract surgical accessibility, with population-based survey data from 55 countries showing a median effective cataract surgical coverage (eCSC) of only 24.8% (2). Coverage varies dramatically from 60.5% in high-income countries to only 14.8% in low-income countries (2). Regional differences are equally pronounced, ranging from 40.4% in Southeast Asia to 13.9% in Africa (2). These coverage gaps represent significant challenges for global health systems, particularly considering that cataract is largely avoidable and treatable. This indicates that cataracts have become a significant global health burden, requiring additional medical resources and preventive measures to effectively address this challenge.

Concurrently, trouble sleeping has emerged as a significant global health concern, affecting approximately one-third of the adult population (3). This condition encompasses various manifestations including obstructive sleep apnea, poor sleep quality, sleep deprivation, insomnia, abnormal sleep duration, and other related conditions. Moreover, it not only impairs quality of life but also potentially increases the risk of various diseases (4), including hypertension (5), diabetes (6), depression (7) and cardiovascular disease (8). A large-scale prospective cohort study in China demonstrated that both short and long sleep duration are associated with increased risk of type 2 diabetes, with optimal sleep duration being 6.3 to 7.5 h per night (9). Importantly, these sleep-related comorbidities are established risk factors for cataract development, creating interconnected pathways that amplify ocular disease risk. Hypertension represents a prominent risk factor in cataract patients, with prevalence reaching 43.8% among posterior subcapsular cataract cases (10). Diabetes contributes through advanced glycation end-products and oxidative stress mechanisms that accelerate lens protein denaturation (11). Depression-related antidepressant use has been significantly associated with increased cataract risk (12). This interplay between sleep disorders and comorbidities establishes convergent pathways that collectively enhance cataract susceptibility.

Evidence suggests that these disorders may influence disease pathogenesis through multiple mechanisms, including circadian rhythm disruption (13), endocrine function alterations (14), and elevated oxidative stress levels (15). Specifically, oxidative stress leads to reactive oxygen species (ROS) accumulation in ocular tissues, which directly contributes to lens opacity formation through oxidative modifications of lens proteins, particularly crystallins, resulting in protein cross-linking and aggregation (16). Additionally, sleep disturbances and circadian rhythm disruption can alter melatonin secretion patterns (17), which may compromise the eye’s natural antioxidant protection, as melatonin serves as a potent scavenger of free radicals and plays a crucial role in maintaining lens transparency (18). Notably, these mechanisms may be amplified in aging populations due to progressive age-related declines in lenticular antioxidant defenses (19) and circulating melatonin levels (20). Beyond age-related physiological changes, contemporary lifestyle factors have further exacerbated these sleep-related health risks, particularly the pervasive use of electronics and social media, which have normalized inadequate sleep patterns among children and adolescents (21). A comprehensive meta-analysis of 55 studies demonstrated that electronic media use is significantly associated with decreased sleep quality and increased sleep problems (22). The COVID-19 pandemic has further highlighted this critical relationship between sleep and health outcomes (21). These findings highlight the critical importance of addressing modifiable lifestyle factors that compromise sleep quality and increase chronic disease susceptibility. Despite these mechanistic insights and growing epidemiological evidence, public awareness of the potential link between sleep health and cataract risk remains limited, underscoring the need for targeted health promotion strategies that emphasize sleep hygiene as a modifiable risk factor in cataract prevention.

To address this knowledge gap and inform such health promotion strategies, recent research has increasingly emphasized the bidirectional and complex relationship between ocular health and sleep disturbances. A systematic scoping review by Choi et al. analyzing 83 studies identified significant associations between 11 eye health conditions (including cataracts, glaucoma, macular degeneration, etc.) and various sleep problems (including insomnia, sleep apnea, circadian rhythm disruptions, etc.), highlighting the important interplay between visual health and sleep quality (23). Furthermore, studies have established that blue light modulates circadian rhythm through intrinsically photosensitive retinal ganglion cells, suggesting a potential mechanistic link with cataract development (24, 25).

The relationship between trouble sleeping and cataracts remains controversial. In a nationwide cohort study, Liu et al. (26) reported a significant association between sleep apnea and increased risk of cataract development. However, Chen et al. (27) in a cross-sectional study of 5,070 participants found no significant association between nuclear cataract and sleep problems after adjusting for potential confounders. These contradictory findings highlight a critical knowledge gap that may stem from differences in study populations (specific sleep apnea vs. general sleep problems), outcome definitions (specific cataract subtypes vs. overall cataract diagnosis), and methodological approaches. Compounding this complexity, emerging evidence suggests sleep duration—a key dimension of sleep disturbance—may independently influence cataract risk. A systematic review and meta-analysis conducted by Zhou et al. (28) revealed a significant association between short sleep duration and cataract risk (OR = 1.20, 95% CI 1.05, 1.36). Similarly, another cross-sectional study found that abnormal sleep duration patterns increased the prevalence risk of cataracts (29). These findings underscore the need to account for sleep duration variations when examining broader sleep-cataract relationships. Our study directly addresses this knowledge gap by providing a population-based analysis using nationally representative data to examine the association between general trouble sleeping and overall cataract diagnosis. This approach offers a broader perspective that complements existing research focused on specific sleep disorders or cataract subtypes, utilizing comprehensive covariate adjustment and robust statistical methodology that accounts for complex sampling design.

## 2 Materials and methods

### 2.1 Study population

This cross-sectional study utilized data from the National Health and Nutrition Examination Survey (NHANES) conducted between 2005 and 2008 in the United States. The selection of this period was necessitated by the concurrent availability of two essential survey modules: the Vision (VIQ) module, which contains the cataract-related outcome variable, was only available in five NHANES cycles spanning 1999–2008; while the Sleep Disorders (SLQ) module, which contains the trouble sleeping exposure variable, has only been available since the 2005–2006 cycle. Consequently, the 2005–2008 cycles represent the sole overlapping period during which both required modules were concurrently available, making this the only feasible data source for directly examining the association between trouble sleeping and cataract within the NHANES framework.

NHANES data are publicly available and were collected under protocols approved by the National Center for Health Statistics Research Ethics Review Board of the Centers for Disease Control and Prevention. As this is a retrospective analysis of de-identified data, informed consent was waived in accordance with the Declaration of Helsinki. The study adhered strictly to ethical guidelines and data use agreements, ensuring participant confidentiality and privacy protection throughout the research process.

**Figure 1:**
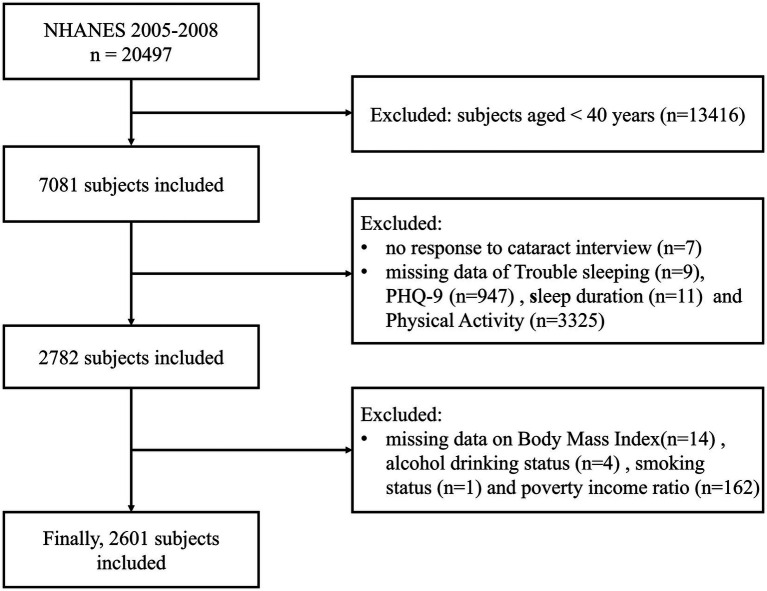
Flowchart for participants recruitment of this study. Data source: National Health and Nutrition Examination Survey (NHANES) 2005–2008.

Initially, 7,081 participants aged ≥40 years were identified. We excluded participants sequentially based on the following criteria: non-response to cataract interview (*n* = 7), lack of data regarding trouble sleeping (*n* = 9), incomplete Patient Health Questionnaire (PHQ) responses (*n* = 947), missing data on other covariates (*n* = 3,517). Finally, a total of 2,601 participants were included in this study (Figure 1).

Additionally, to assess the potential impact of missing data on our findings, we conducted a comprehensive comparison between excluded and included participants across all baseline characteristics. This analysis examined differences in demographic factors, lifestyle variables, and comorbidities to identify potential selection bias patterns. The results of this missing data analysis are presented in Supplementary Table S2.

### 2.2 Definition of trouble sleeping and cataracts

According to the third edition of the American Academy of Sleep Medicine’s classification of sleep disorders, trouble sleeping is defined as a persistent difficulty with sleep initiation, duration, consolidation, or quality that occurs although acquiring adequate opportunity and circumstances for sleeping, and results in some form of daytime impairment (30). Trouble sleeping contained obstructive sleep apnea, sleep quality complaints (sleep deprivation, sleep duration and insomnia), with a combination of other sleep problems (31). Trouble sleeping was assessed through the following question: “Have you ever told a doctor or other health professional that you have trouble sleeping.” Those who responded “yes” were classified as having trouble sleeping.

Cataract status was determined through the question “Have you ever had a cataract operation?” Those who answered “yes” were considered to have cataract.

### 2.3 Covariates

We included the following covariates: (1) demographic characteristics: age, gender, race/ethnicity (categorized as Mexican American, Other Hispanic, Non-Hispanic White, Non-Hispanic Black, Other Race), education level (categorized as Less than High School, High School Graduate, College or Above), marital status (Married/Living with partner, Divorced/Separated/ Widowed, Never Married), and poverty income ratio (PIR). PIR was graded into three categories based on the analysis guideline: ≤1.3, 1.3–3.5, and >3.5 (32). (2) Lifestyle factors: body mass index (BMI), sleep duration (hours), alcohol drinking status (yes/no), smoking status (yes/no), and physical activity. Alcohol drinking status was determined by the question “In any one year, have you had at least 12 drinks of any type of alcoholic beverage?” Smoking status was assessed through the question “Have you smoked at least 100 cigarettes in your entire life?” Physical activity data were obtained from household interviews, and leisure-time physical activity over the past 30 days was used to calculate weekly metabolic equivalents (MET). MET scores = weekly frequency of each physical activity * duration of each physical activity* each physical activity suggested MET Scores. (3) Comorbidities: hypertension, diabetes, kidney disease, high cholesterol, coronary heart disease (CHD), stroke, and depression (all yes/no). Information on these conditions was collected via self-reported questionnaires. Chronic conditions were defined through affirmative responses to specific NHANES questionnaire items: hypertension (BPQ020), diabetes (DIQ010), kidney disease (KIQ022), high cholesterol (BPQ080), coronary heart disease (MCQ160C), and stroke (MCQ160F). These items uniformly assess physician-diagnosed conditions using the question framework ‘Has a doctor ever told you that you had [condition]?’ Depressive symptoms were evaluated using the Patient Health Questionnaire (PHQ), a self-report measure consisting of 9 signs and symptoms. Each item is scored from 0 to 3, with total scores ranging from 0 to 27. Based on previous research (33), scores ≥10 were considered indicative of depression.

### 2.4 Statistical analysis

All statistical analyses were performed using R version 4.4.1, incorporating survey weights with a 1/2 scaling factor applied to WTMEC2YR per National Center for Health Statistics (NCHS) guidelines for combined cycles, and accounting for the complex sampling design to ensure nationally representative estimates. For descriptive analyses, continuous variables were presented as survey-weighted means with 95% confidence intervals using survey-weighted linear regression (svyglm), while categorical variables were expressed as survey-weighted percentages with 95% confidence intervals. Between-group comparisons were conducted using survey-weighted linear regression for continuous variables and survey-weighted Chi-square tests (svytable) for categorical variables. The association between trouble sleeping and cataract was examined using survey-weighted logistic regression models with progressive adjustment for potential confounders. Four sequential models were constructed following established epidemiological principles for confounder adjustment: Model 1 (unadjusted baseline association); Model 2 (adjusted for sociodemographic factors including age, gender, race/ethnicity, education level, marital status, and PIR, as these demographic characteristics are fundamental confounders that influence both sleep patterns and cataract risk); Model 3 (further adjusted for lifestyle factors including BMI, sleep duration, alcohol drinking status, smoking status, and physical activity, as these modifiable behavioral factors represent intermediate pathways that may mediate the sleep-cataract relationship); and Model 4 (fully adjusted model additionally including comorbidities such as hypertension, diabetes, kidney disease, high cholesterol, CHD, stroke, and depression, as these chronic conditions share common risk factors with both sleep disorders and cataract development, potentially acting as confounders or mediators in the association). Stratified analyses were performed to assess potential effect modification by key demographic and clinical characteristics, with interaction terms tested in the fully adjusted model. Results were presented as odds ratios (ORs) with 95% confidence intervals. Two-sided *p*-values < 0.05 were considered statistically significant. Prior to logistic regression modeling, multicollinearity among all covariates was systematically assessed using generalized variance inflation factors (GVIF) in the context of our complex survey design. Additionally, correlation matrices among continuous variables were examined to identify potential problematic associations. Detailed multicollinearity assessment results are presented in Supplementary Tables S3 and S4. Post-hoc statistical power analysis was conducted to validate sample size adequacy using the standard error derived from the 95% confidence interval of the main association.

## 3 Result

### 3.1 Demographic characteristics of the study sample

Baseline characteristics of study participants are presented in Table 1. Compared with those without cataracts, participants with cataracts were significantly older (71.2 vs. 54.0 years, *p* < 0.0001) and more likely to be Non-Hispanic White (88.9% vs. 80.1%, *p* = 0.0047). They demonstrated lower educational attainment (college or above: 53.3% vs. 68.7%, *p* < 0.0001) and were more frequently in middle-income categories (PIR 1.3–3.5: 52.7% vs. 28.6%, *p* < 0.0001). Regarding lifestyle factors, the cataract group showed higher smoking rates (56.0% vs. 47.1%, *p* = 0.0154), lower physical activity (1,180.6 vs. 1,500.2 MET-minutes/week, *p* = 0.0195), and more frequent trouble sleeping (41.3% vs. 26.0%, *p* < 0.0001). Notably, the prevalence of trouble sleeping was substantially higher among participants with cataracts compared to those without cataracts, with an absolute difference of 15.3 percentage points, representing a 1.6-fold higher prevalence in the cataract group. Additionally, participants with cataracts exhibited significantly higher prevalence of comorbidities, including hypertension (59.2% vs. 37.3%, *p* < 0.0001), high cholesterol (53.4% vs. 40.6%, *p* = 0.0011), diabetes (16.3% vs. 7.5%, *p* < 0.0001), coronary heart disease (11.8% vs. 3.9%, *p* < 0.0001), and stroke (8.3% vs. 1.9%, *p* < 0.0001).

**Table 1:** Baseline characteristics of the study participants.

Characteristics	Without cataract	Cataract	*p*-value
Age, years	54.0 (53.0, 54.9)	71.2 (69.8, 72.5)	<0.0001
Gender			0.0694
Male	49.5 (46.9, 52.1)	43.2 (35.9, 50.8)	
Female	50.5 (47.9, 53.1)	56.8 (49.2, 64.1)	
Race/ethnicity			0.0047
Mexican American	3.9 (3.2, 4.8)	2.0 (1.0, 4.0)	
Other Hispanic	2.6 (1.7, 3.9)	1.4 (0.5, 3.6)	
Non-Hispanic white	80.1 (76.0, 83.6)	88.9 (84.6, 92.1)	
Non-Hispanic black	9.0 (6.6, 12.0)	4.8 (3.1, 7.5)	
Other race	4.4 (3.2, 6.0)	2.9 (1.1, 7.2)	
Education level			<0.0001
Less than high school	9.9 (8.1, 11.9)	18.3 (14.2, 23.4)	
High school graduate	21.5 (19.3, 23.8)	28.4 (22.9, 34.6)	
College or above	68.7 (65.1, 72.0)	53.3 (44.8, 61.5)	
PIR			<0.0001
≤1.3	9.9 (8.1, 12.2)	11.8 (7.8, 17.5)	
1.3–3.5	28.6 (25.1, 32.5)	52.7 (46.1, 59.3)	
>3.5	61.4 (56.6, 66.0)	35.5 (28.7, 42.9)	
Marital status			<0.0001
Married/living with partner	72.4 (69.2, 75.3)	60.5 (50.6, 69.7)	
Divorced/separated/widowed	21.3 (18.9, 23.9)	37.3 (28.5, 47.1)	
Never married	6.4 (4.9, 8.2)	2.1 (0.7, 6.1)	
Alcohol drinking status			0.0862
Yes	75.8 (72.0, 79.3)	70.4 (62.6, 77.2)	
No	24.2 (20.7, 28.0)	29.6 (22.8, 37.4)	
Smoking status			0.0154
Yes	47.1 (44.1, 50.1)	56.0 (48.4, 63.4)	
No	52.9 (49.9, 55.9)	44.0 (36.6, 51.6)	
BMI (kg/m^2^)	28.6 (28.3, 28.9)	27.8 (27.1, 28.4)	0.0233
Physical activity (MET) (minute/week)	1,500.2 (1,401.8, 1,598.6)	1,180.6 (940.6, 1,420.6)	0.0195
Sleep duration (hours)	6.9 (6.9, 7.0)	7.1 (6.9, 7.3)	0.1840
Depression			0.6419
Yes	3.3 (2.4, 4.4)	2.6 (1.0, 6.5)	
No	96.7 (95.6, 97.6)	97.4 (93.5, 99.0)	
Trouble sleeping			<0.0001
Yes	26.0 (23.8, 28.3)	41.3 (34.3, 48.7)	
No	74.0 (71.7, 76.2)	58.7 (51.3, 65.7)	
Stroke			<0.0001
Yes	1.9 (1.3, 2.7)	8.3 (5.4, 12.6)	
No	98.1 (97.3, 98.7)	91.7 (87.4, 94.6)	
CHD			<0.0001
Yes	3.9 (2.9, 5.2)	11.8 (8.5, 16.1)	
No	96.1 (94.8, 97.1)	88.2 (83.9, 91.5)	
Hypertension			<0.0001
Yes	37.3 (34.0, 40.8)	59.2 (53.6, 64.7)	
No	62.7 (59.2, 66.0)	40.8 (35.3, 46.4)	
High cholesterol			0.0011
Yes	40.6 (37.4, 43.9)	53.4 (47.3, 59.4)	
No	59.4 (56.1, 62.6)	46.6 (40.6, 52.7)	
Diabetes			<0.0001
Yes	7.5 (6.2, 9.0)	16.3 (12.7, 20.7)	
No	92.5 (91.0, 93.8)	83.7 (79.3, 87.3)	
Kidney disease			0.1257
Yes	1.5 (1.0, 2.2)	2.9 (1.3, 6.6)	
No	98.5 (97.8, 99.0)	97.1 (93.4, 98.7)	

For continuous variables: survey-weighted mean (95% CI), p-value was by survey-weighted linear regression (svyglm); For categorical variables: survey-weighted percentage (95% CI), p-value was by survey-weighted Chi-square test (svytable). PIR, poverty income ratio. BMI, body mass index. CHD, coronary heart disease. Source: National Health and Nutrition Examination Survey (NHANES) 2005–2008.

### 3.2 Missing data analysis

Comparison between excluded (*n* = 4,480) and included (*n* = 2,601) participants revealed several demographic and clinical differences (Supplementary Table S2). Excluded participants were significantly older (61.9 ± 13.2 vs. 58.4 ± 12.3 years, *p* < 0.001), had higher BMI (29.4 ± 6.9 vs. 28.8 ± 5.8 kg/m^2^, *p* < 0.001), and showed higher prevalence of multiple comorbidities including diabetes (20.0% vs. 11.8%, *p* < 0.001), hypertension (48.4% vs. 43.6%, *p* < 0.001), depression (13.0% vs. 3.7%, *p* < 0.001), coronary heart disease (6.9% vs. 5.3%, *p* = 0.006), and stroke (7.7% vs. 3.5%, *p* < 0.001). However, the primary exposure variable (trouble sleeping) showed no significant difference between groups (27.5% vs. 26.0%, *p* = 0.176). Excluded participants had significantly higher cataract prevalence (17.4% vs. 10.7%, *p* < 0.001). Sleep duration also differed between groups (7.1 ± 5.5 vs. 6.9 ± 1.3 h, *p* = 0.011), though the absolute difference was small (0.2 h).

### 3.3 Association between trouble sleeping and cataract risk

In this study, the association between trouble sleeping and cataract was examined through a series of increasingly adjusted logistic regression models (Table 2). The unadjusted model showed that participants with trouble sleeping had approximately twice the odds of having cataracts compared to those without trouble sleeping (OR = 2.01, 95% CI: 1.42–2.83, *p* < 0.001). After adjusting for sociodemographic factors, the association strengthened (OR = 2.66, 95% CI: 1.71–4.15, *p* < 0.001). Further adjustment for lifestyle factors resulted in a slight attenuation of the association (OR = 2.59, 95% CI: 1.55–4.34, *p* = 0.001). In the fully adjusted model, which accounted for various comorbidities, the association remained robust and clinically significant (OR = 2.50, 95% CI: 1.35–4.62, *p* = 0.011), indicating that individuals with trouble sleeping had 2.5 times higher odds of having cataracts, independent of potential confounding factors. Post-hoc power analysis confirmed that our study achieved 83.1% statistical power to detect the observed association, substantially exceeding the conventional 80% threshold and validating the adequacy of our sample size for detecting clinically meaningful associations between trouble sleeping and cataracts.

**Table 2:** Logistic regression models for trouble sleeping and cataract.

Models	OR (95% CI)	*p*-value
Model 1	2.01 (1.42, 2.83)	<0.001
Model 2	2.66 (1.71, 4.15)	<0.001
Model 3	2.59 (1.55, 4.34)	0.001
Model 4	2.50 (1.35, 4.62)	0.011

OR, odds ratio; CI, confidence interval; PIR, poverty income ratio; BMI, body mass index; CHD, coronary heart disease. Model 1: Unadjusted. Model 2: Adjusted for sociodemographic factors. Model 3: Additionally adjusted for lifestyle factors. Model 4: Fully adjusted model including comorbidities. Source: National Health and Nutrition Examination Survey (NHANES) 2005–2008.

### 3.4 Subgroup analyses

**Figure 2:**
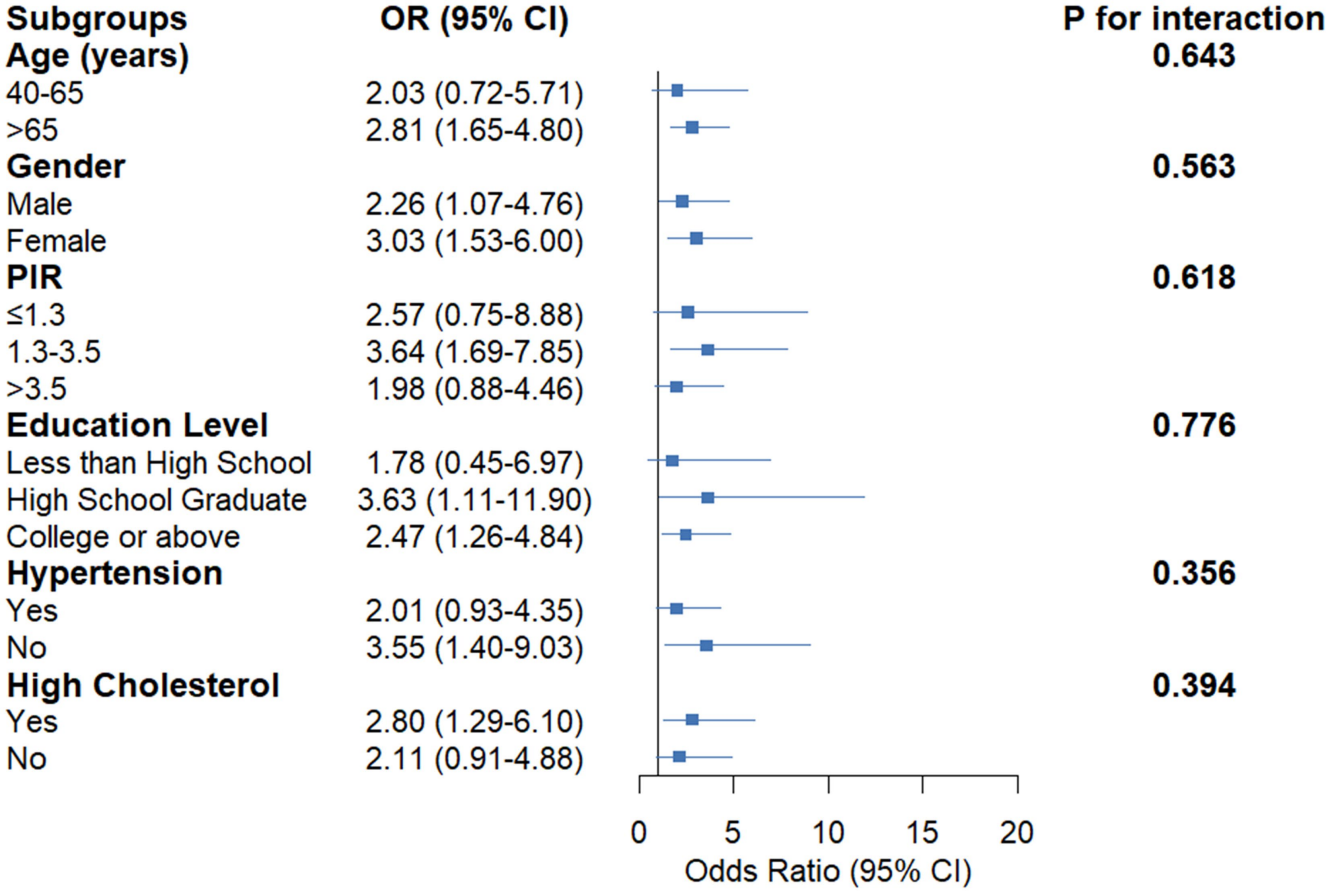
Forest plot showing the association between trouble sleeping and cataract prevalence stratified by demographic and clinical characteristics. Model was adjusted for all covariates. OR, odds ratio; CI, confidence interval; PIR, poverty income ratio. Data source: National Health and Nutrition Examination Survey (NHANES) 2005–2008.

Stratified analyses were conducted to explore the potential effect modification by demographic and clinical characteristics in the association between trouble sleeping and cataract (Figure 2). The results revealed consistent positive associations across all subgroups, with odds ratios ranging from 1.78 to 3.64. The association between trouble sleeping and cataract remained statistically significant across multiple demographic subgroups, including individuals aged >65 years (OR = 2.81, 95% CI: 1.65–4.80, *p* = 0.003), females (OR = 3.03, 95% CI: 1.53–6.00, *p* = 0.006), those with PIR 1.3–3.5 (OR = 3.64, 95% CI: 1.69–7.85, *p* = 0.005), and high school graduates (OR = 3.63, 95% CI: 1.11–11.90, *p* = 0.039). The association also remained significant regardless of the presence or absence of comorbidities such as hypertension and high cholesterol. Importantly, the interaction analyses revealed no significant effect modification by age (*p* for interaction = 0.643), gender (*p* = 0.563), PIR (*p* = 0.618), education level (*p* = 0.776), hypertension status (*p* = 0.356), or high cholesterol status (*p* = 0.394), indicating that the strength of association between trouble sleeping and cataract is consistent across these demographic and clinical subgroups.

### 3.5 Sensitivity analyses

The missing covariate data were processed through multiple imputation methodology, yielding five complete data versions that were subsequently integrated during the final analytical phase. The results further confirmed a consistent and robust positive association between trouble sleeping and cataract (OR: 2.50; 95% CI: 1.52–4.10) (Supplementary Table S1).

## 4 Discussion

In this cross-sectional study utilizing nationally representative NHANES data from 2005 to 2008, we investigated the association between trouble sleeping and cataract among 2,601 US adults aged ≥40 years. Our analysis revealed that individuals with trouble sleeping had significantly higher odds of having cataracts (OR = 2.50, 95% CI: 1.35–4.62, *p* = 0.011). This association remained robust across various demographic and clinical subgroups, with interaction tests indicating no significant differences between subgroups, demonstrating the consistency of this association across diverse populations.

Trouble sleeping are closely associated with various ophthalmic conditions, including glaucoma (34–36), low vision (37), age-related macular degeneration (38, 39), and diabetic retinopathy (40). These ocular diseases demonstrate a positive correlation with deteriorating sleep quality. Furthermore, the relationship between trouble sleeping and cataract has been previously proposed and investigated. A large-scale nationwide cohort study conducted by Liu et al. (26). Demonstrated that patients experiencing sleep apnea showed a significantly higher risk of developing cataracts (adjusted hazard ratio [95% CI]: 1.4 [1.2–1.6]). This association might be attributed to repetitive hypoxia-reoxygenation cycles leading to oxidative stress and systemic inflammatory responses. Our findings align with their results, revealing that individuals with trouble sleeping in the U.S. adult population face a significantly increased risk of cataracts. Furthermore, recognizing sleep duration as an established risk factor for cataract development (as detailed in the Introduction), we rigorously adjusted for this potential confounder in logistic regression model 3, and the association between trouble sleeping and cataracts remained significant (OR = 2.59, 95% CI: 1.55–4.34, *p* = 0.001).

Sleep abnormalities (trouble sleeping or sleeping too much) are included among the nine questions in the Patient Health Questionnaire used for depression assessment. Previous studies have also demonstrated an association between sleep disorders and the occurrence of depression (41, 42). Therefore, we adjusted for depression as a covariate in regression model 4, and found that the association between trouble sleeping and cataracts remained robust (OR = 2.50, 95% CI: 1.35–4.62, *p* = 0.011). This further strengthens the reliability of our findings. Interestingly, however, some studies have reported different results. Chen et al. (27) employed a standardized protocol for nuclear cataract grading and found no statistically significant association between sleep problem and the presence of either nuclear cataract or intraocular lens. Similarly, Obayashi et al. (43) observed that while cataract surgery groups exhibited differences in certain objective sleep parameters, no significant differences were found in sleep onset latency, total sleep time, and sleep-mid time compared to non-surgery groups. The observed discrepancies in findings could be attributed to differences in study design, population characteristics, and operational definitions of sleep problems.

Furthermore, our analysis demonstrated that the association between trouble sleeping and cataract was consistent across demographic subgroups. While statistically significant associations were observed in various subgroups including individuals aged over 65 years and females, the interaction tests revealed no significant differences between subgroups, indicating that the association is robust across different demographic characteristics. Although interaction testing showed no statistically significant effect modification by sex (*p* = 0.563), the point estimates suggested a numerically stronger association in females (OR = 3.03, 95% CI: 1.53–6.00) compared to males (OR = 1.71, 95% CI: 0.85–3.43). Several biological and epidemiological factors may help explain this observed pattern. This finding aligns with established gender disparities: women exhibit 41% higher insomnia prevalence than men (44) and bear disproportionate cataract burden, with age-standardized blindness prevalence of 0.19% versus 0.13% in males (*p* < 0.001) (45). Sex hormones, particularly estrogen and progesterone, may mediate these differences through regulatory effects on circadian rhythms (46), sleep architecture (47), and antioxidant defenses. Postmenopausal estrogen decline could amplify both sleep disturbances and cataract susceptibility, warranting investigation of hormone dynamics as potential moderators of this relationship. The biological mechanism underlying this association may involve circadian rhythm disruption and melatonin secretion. Studies demonstrated that disrupted sleep patterns can alter melatonin secretion, which plays a crucial role in lens protection against oxidative stress (18, 48). Additionally, studies have shown that cataract surgery can improve melatonin secretion patterns and subsequent sleep quality, suggesting a bidirectional relationship between lens opacity and sleep regulation (49).

This study demonstrates several notable strengths. Firstly, it utilizes the NHANES database, a large-scale national dataset, employing rigorous sampling design and weighted analysis methods to ensure result representativeness and generalizability. Secondly, the study implements a systematic data analysis strategy through four progressively adjusted regression models, comprehensively controlling for potential confounders including demographic characteristics, lifestyle factors, and comorbidities, thereby enhancing the reliability of findings. Thirdly, detailed stratified analyses were conducted to examine the association between trouble sleeping and cataracts across different subgroups of age, gender, and income levels, providing more refined clinical reference points. Fourthly, our missing data analysis revealed that while excluded participants differed in some characteristics, the primary exposure variable (trouble sleeping) was similarly distributed between excluded and included groups (27.5% vs. 26.0%, *p* = 0.176), minimizing concerns about selection bias for our main hypothesis. Importantly, excluded participants had higher cataract prevalence (17.4% vs. 10.7%, *p* < 0.001), creating a conservative bias that would attenuate rather than inflate our observed associations, thus strengthening the credibility of our findings. Additionally, the study employed standardized assessment tools and stringent statistical methods, including the PHQ-9 scale for depression evaluation and weighted analysis under complex sampling design, further strengthening the scientific rigor and credibility of the research findings. Furthermore, our post-hoc power analysis demonstrated that the study achieved 83.1% statistical power to detect the observed association (OR = 2.50, 95% CI: 1.35–4.62), confirming adequate sample size and supporting the reliability of our findings. This power level exceeds Cohen’s conventional 80% threshold, indicating robust statistical adequacy for detecting clinically meaningful associations. The power calculation methodology, based on the actual standard error derived from our confidence intervals, inherently accounts for the complex NHANES sampling design and provides a conservative estimate of our study’s statistical capability.

### 4.1 Limitations of the current study

However, this study has several noteworthy limitations. First, although NHANES data are nationally representative, the study only included data from 2005 to 2008, which may limit generalizability to current populations. Over the past 15–20 years, significant changes in sleep medicine awareness, cataract surgical techniques, population health characteristics, and healthcare access may have altered both the prevalence of these conditions and their relationship. These temporal changes underscore the need for validation studies using contemporary data to confirm the current relevance of our findings. Second, and most critically, the present study’s complete reliance on questionnaire-based, self-reported data for the assessment of both key variables represents a fundamental methodological constraint that renders the conclusions of this study preliminary rather than definitive. In clinical and research practice, the gold standard for objective sleep assessment is overnight polysomnography (PSG), which integrates electroencephalography (EEG), electrooculography (EOG), and electromyography (EMG) alongside respiratory and oximetric signals to enable precise quantification of sleep architecture including N1, N2, N3 slow-wave sleep, and REM sleep stages, as well as accurate measurement of sleep onset latency, sleep efficiency, total sleep time, arousal index, and apnea-hypopnea index. The single-item self-report approach employed in the present study is susceptible to recall bias, social desirability bias, and healthcare-access bias—individuals who have never consulted a clinician about sleep difficulties may harbor undiagnosed sleep disorders, thereby systematically underestimating the true prevalence of sleep disturbances in the study population. Furthermore, this item cannot differentiate between distinct sleep disorder subtypes (e.g., obstructive sleep apnea, insomnia disorder, circadian rhythm disorder) or quantify severity, precluding any dose–response analysis. The resulting exposure misclassification is likely non-differential in direction, tending to bias the observed association toward the null; however, differential misclassification cannot be entirely excluded. Similarly, cataract status was ascertained through a single self-reported question—“Have you ever had a cataract operation?”—rather than through objective ophthalmic evaluation. A comprehensive clinical assessment of cataract centers on slit-lamp biomicroscopy, supplemented by retroillumination photography for objective image documentation, with the Lens Opacities Classification System III (LOCS III) applied to standardize grading of slit-lamp and retroillumination findings, thereby enabling systematic identification and quantification of the three major cataract subtypes—nuclear, cortical, and posterior subcapsular—as well as pre-symptomatic or early-stage lens opacities that have not yet necessitated surgical intervention. Defining cataract status solely on the basis of prior surgical history introduces significant ascertainment bias: it systematically excludes individuals with clinically significant but surgically untreated cataracts. Consequently, the true burden of cataract in the study population is likely substantially underestimated. Taken together, the dual reliance on single-item subjective self-reporting for both the exposure and outcome variables substantially limits the precision and validity of the observed association, and the reported findings should therefore be interpreted with appropriate caution. Third, the cross-sectional design of this study precludes any inference regarding the temporal relationship between trouble sleeping and cataract development. It remains uncertain whether sleep disorders precede and contribute to cataract formation through chronic oxidative stress and disrupted melatonin-mediated antioxidant protection of the lens, or conversely, whether progressive visual impairment due to lens opacity disrupts photic input to the suprachiasmatic nucleus via intrinsically photosensitive retinal ganglion cells, thereby secondarily impairing circadian regulation and sleep quality. The bidirectional nature of this potential relationship has been highlighted by evidence that cataract surgery can improve melatonin secretion and sleep quality, suggesting that cataracts themselves may contribute to sleep disruption. Without longitudinal data with clearly established temporal sequencing of exposure and outcome, causal directionality cannot be determined, and the conclusions of this study must therefore be regarded as hypothesis-generating rather than confirmatory. Fourth, while the study adjusted for multiple known confounding factors, there might be unmeasured confounders, such as environmental and genetic factors, that could affect the interpretation of results. Future prospective cohort studies incorporating objective sleep assessment and standardized ophthalmic evaluation are strongly warranted to overcome these limitations, to establish the temporal directionality of the sleep-cataract relationship, and to elucidate the underlying biological mechanisms.

Given the significant association between trouble sleeping and cataract risk identified in our study, several clinical implications emerge for both ophthalmology and primary care practice. First, routine sleep assessment should be integrated into comprehensive eye examinations, particularly for patients over 40 years of age who are at increased risk for cataract development. Ophthalmologists could incorporate standardized sleep questionnaires such as the Pittsburgh Sleep Quality Index (PSQI) or the Epworth Sleepiness Scale into their patient evaluation protocols. Second, primary care providers should consider ocular health screening for patients presenting with chronic sleep disorders, as our findings suggest these individuals may be at elevated risk for cataract development. Third, interdisciplinary collaboration between ophthalmologists, sleep specialists, and primary care physicians could optimize patient care by addressing both sleep health and ocular health simultaneously. Fourth, patient education programs should emphasize the bidirectional relationship between sleep quality and eye health, encouraging patients to report sleep disturbances during routine eye examinations and to seek appropriate sleep disorder treatment when indicated. From a sleep medicine perspective, ophthalmologic evaluation may serve as an opportune entry point for detecting undiagnosed sleep disorders, particularly given that patients seeking cataract care often present with established risk factors for sleep disturbances (advanced age, comorbidities such as hypertension and diabetes). This bidirectional screening approach could enhance early detection of both conditions, enabling timely intervention that may improve outcomes for both ocular health and sleep quality, ultimately reducing the dual burden of these conditions on public health systems. Beyond screening and risk stratification, several potential interventions merit consideration for both sleep health optimization and cataract prevention, though longitudinal studies are needed to establish their efficacy. Sleep hygiene interventions represent a foundational approach, including structured sleep–wake schedules, reduction of pre-bedtime electronic device exposure (which addresses both blue light-induced circadian disruption and sleep quality degradation), and optimization of sleep environment factors such as bedroom temperature, lighting, and noise control. For patients with diagnosed sleep disorders, targeted treatments such as continuous positive airway pressure (CPAP) therapy for obstructive sleep apnea may confer dual benefits by reducing systemic oxidative stress—a shared pathological mechanism in both sleep apnea and cataractogenesis. Additionally, lifestyle modifications including antioxidant-rich dietary patterns (e.g., increased intake of vitamins C and E, lutein, and zeaxanthin) and regular moderate-intensity physical activity have been independently associated with both improved sleep quality and reduced cataract risk through antioxidant and anti-inflammatory pathways. From a preventive medicine perspective, prioritized cataract evaluations for patients with chronic sleep disorders could enable earlier detection and timely intervention. Finally, healthcare systems could implement screening protocols that identify high-risk patients with both sleep disorders and age-related eye disease risk factors, enabling early intervention and potentially reducing the burden of cataract-related visual impairment. However, these recommendations should be considered preliminary, as our cross-sectional design cannot establish causality, and prospective intervention trials are needed to confirm whether sleep-targeted interventions could effectively reduce cataract risk.

## 5 Conclusion

This study, based on NHANES 2005–2008 data, found that trouble sleeping was significantly associated with higher odds of cataracts, even after adjusting for confounders. Interaction analyses confirmed no significant effect modification by demographic or clinical characteristics, indicating the robustness of this association across diverse populations. Our findings suggest that incorporating sleep health considerations into public health strategies may have potential value for cataract prevention and overall ocular health improvement. Future research should focus on longitudinal studies to establish causality, explore the biological mechanisms linking trouble sleeping to cataract development, and investigate whether sleep interventions could reduce cataract risk.

## Data Availability

The datasets presented in this study can be found in online repositories. The names of the repository/repositories and accession number(s) can be found at: the datasets generated and analyzed during the current study are available in the NHANES repository (https://www.cdc.gov/nchs/nhanes/).
