# Supplementary material for: Association between trouble sleeping and cataract in US adults: a cross-sectional study
Source: Front Med (Lausanne). 2026 Apr 23;13:1535667. doi: 10.3389/fmed.2026.1535667 (PMC13149138; doi:10.3389/fmed.2026.1535667)
Supplement: Supplementary file 1 [file Table_1.DOC]

Table S1 Logistic Regression Models for trouble sleeping and cataract after multiple imputation of missing covariates

| **Models** | **OR (95% CI)** | **P-value** |
| --- | --- | --- |
| Model 1 | 2.01 (1.44, 2.79) | <0.001 |
| Model 2 | 2.66 (1.76, 3.99) | <0.001 |
| Model 3 | 2.59 (1.62, 4.13) | 0.002 |
| Model 4 | 2.50 (1.52, 4.10) | 0.017 |

OR, odds ratio; CI, confidence interval; PIR, poverty income ratio; BMI, body mass index; CHD, coronary heart disease.

Model 1: Unadjusted

Model 2: Adjusted for age, gender, race/ethnicity, education level, marital status, and PIR

Model 3: Adjusted for the variables in Model 2, plus BMI, Sleep Duration, Alcohol drinking status, Smoking status, and Physical Activity.

Model 4: Adjusted for the variables in Model 3, plus Hypertension, Diabetes, Kidney Disease, High Cholesterol, CHD, Stroke, and Depression.
