# Supplementary material for: Association between trouble sleeping and cataract in US adults: a cross-sectional study
Source: Front Med (Lausanne). 2026 Apr 23;13:1535667. doi: 10.3389/fmed.2026.1535667 (PMC13149138; doi:10.3389/fmed.2026.1535667)
Supplement: Supplementary file 2 [file Table_2.DOC]

Table S2 Baseline Characteristics Comparison Between Excluded and Included Participants

| **Characteristic** | **Excluded Group (n=4,480)** | **Included Group (n=2,601)** | **P-value** |
| --- | --- | --- | --- |
| Age (mean ± SD) | 61.9 ± 13.2 | 58.4 ± 12.3 | <0.001 |
| BMI (mean ± SD) | 29.4 ± 6.9 | 28.8 ± 5.8 | <0.001 |
| Sleep Duration hours (mean ± SD) | 7.1 ± 5.5 | 6.9 ± 1.3 | 0.011 |
| Physical Activity (mean ± SD) | 1554.4 ± 3235.5 | 1448.8 ± 1917.3 | 0.471 |
| Female (%) | 48.1 | 52.0 | 0.002 |
| Trouble Sleeping (%) | 27.5 | 26.0 | 0.176 |
| Alcohol Drinking (%) | 64.3 | 71.4 | <0.001 |
| Smoking (%) | 52.7 | 49.4 | 0.009 |
| Diabetes (%) | 20.0 | 11.8 | <0.001 |
| Hypertension (%) | 48.4 | 43.6 | <0.001 |
| Cholesterol (%) | 51.2 | 48.6 | 0.055 |
| Depression (%) | 13.0 | 3.7 | <0.001 |
| CHD (%) | 6.9 | 5.3 | 0.006 |
| Stroke (%) | 7.7 | 3.5 | <0.001 |
| Cataract (%) | 17.4 | 10.7 | <0.001 |

BMI, body mass index; CHD, coronary heart disease.
