# Supplementary material for: Association between trouble sleeping and cataract in US adults: a cross-sectional study
Source: Front Med (Lausanne). 2026 Apr 23;13:1535667. doi: 10.3389/fmed.2026.1535667 (PMC13149138; doi:10.3389/fmed.2026.1535667)
Supplement: Supplementary file 3 [file Table_3.DOC]

Table S3 Multicollinearity Assessment Among Covariates Using Generalized Variance Inflation Factors (GVIF)

| **Variable** | **GVIF** | **Df** | **Adjusted GVIF^(1/(2×Df))** |
| --- | --- | --- | --- |
| Age (years) | 1.316 | 1 | 1.147 |
| Gender | 1.295 | 1 | 1.138 |
| Race/ethnicity | 1.416 | 4 | 1.044 |
| Education level | 1.412 | 2 | 1.090 |
| Poverty income ratio | 1.409 | 2 | 1.089 |
| Marital status | 1.196 | 2 | 1.046 |
| Body mass index | 1.167 | 1 | 1.080 |
| Sleep duration | 1.158 | 1 | 1.076 |
| Alcohol consumption | 1.223 | 1 | 1.106 |
| Smoking status | 1.159 | 1 | 1.077 |
| Physical activity | 1.036 | 1 | 1.018 |
| Coronary heart disease | 1.098 | 1 | 1.048 |
| Stroke | 1.055 | 1 | 1.027 |
| Depression | 1.080 | 1 | 1.039 |
| Hypertension | 1.113 | 1 | 1.055 |
| High cholesterol | 1.053 | 1 | 1.026 |
| Diabetes | 1.132 | 1 | 1.064 |
| Kidney disease | 1.034 | 1 | 1.017 |

GVIF, Generalized Variance Inflation Factor; Df, degrees of freedom. Adjusted GVIF values are calculated as GVIF^(1/(2×Df)) to account for different degrees of freedom among variables.
