# Supplementary material for: Association between trouble sleeping and cataract in US adults: a cross-sectional study
Source: Front Med (Lausanne). 2026 Apr 23;13:1535667. doi: 10.3389/fmed.2026.1535667 (PMC13149138; doi:10.3389/fmed.2026.1535667)
Supplement: Supplementary file 4 [file Table_4.DOC]

Table S4 Correlation Matrix Among Continuous Variables

| **Variable** | **Age (RIDAGEYR)** | **BMI (BMXBMI)** | **Sleep Duration** | **Physical Activity** |
| --- | --- | --- | --- | --- |
| **Age (RIDAGEYR)** | 1.000 | -0.115 | 0.162 | -0.079 |
| **BMI (BMXBMI)** | -0.115 | 1.000 | -0.068 | -0.068 |
| **Sleep Duration** | 0.162 | -0.068 | 1.000 | -0.017 |
| **Physical Activity** | -0.079 | -0.068 | -0.017 | 1.000 |

BMI, body mass index; Values represent Pearson correlation coefficients used to assess multicollinearity among continuous variables.
